# Interaction between Angiotensin Type 1, Type 2, and Mas Receptors to Regulate Adult Neurogenesis in the Brain Ventricular–Subventricular Zone

**DOI:** 10.3390/cells8121551

**Published:** 2019-11-30

**Authors:** Maria Garcia-Garrote, Ana Perez-Villalba, Pablo Garrido-Gil, German Belenguer, Juan A. Parga, Francisco Perez-Sanchez, Jose Luis Labandeira-Garcia, Isabel Fariñas, Jannette Rodriguez-Pallares

**Affiliations:** 1 Laboratorio de Neurobiología Celular y Molecular de la Enfermedad de Parkinson, Centro Singular de Investigación en Medicina Molecular y Enfermedades Crónicas (CiMUS), Dpto. Ciencias Morfolóxicas, Universidade de Santiago de Compostela, 15782 Santiago de Compostela, Spain; 2 Centro de Investigación Biomédica en Red de Enfermedades Neurodegenerativas (CIBERNED), 28031 Madrid, Spain; 3 Faculty of Psychology, Universidad Católica de Valencia, Valencia, 46100 Burjassot, Spain; 4 Departamento de Biología Celular, Biología Funcional y Antropología Física and Estructura de Recerca Interdisciplinar en Biotecnologia i Biomedicina (ERI BIOTECMED), Universidad de Valencia, 46100 Burjassot, Spain

**Keywords:** neural stem cells, ventricular–subventricular zone, AT1 receptors, AT2 receptors, aging, proliferation, neurospheres

## Abstract

The renin–angiotensin system (RAS), and particularly its angiotensin type-2 receptors (AT2), have been classically involved in processes of cell proliferation and maturation during development. However, the potential role of RAS in adult neurogenesis in the ventricular-subventricular zone (V-SVZ) and its aging-related alterations have not been investigated. In the present study, we analyzed the role of major RAS receptors on neurogenesis in the V-SVZ of adult mice and rats. In mice, we showed that the increase in proliferation of cells in this neurogenic niche was induced by activation of AT2 receptors but depended partially on the AT2-dependent antagonism of AT1 receptor expression, which restricted proliferation. Furthermore, we observed a functional dependence of AT2 receptor actions on Mas receptors. In rats, where the levels of the AT1 relative to those of AT2 receptor are much lower, pharmacological inhibition of the AT1 receptor alone was sufficient in increasing AT2 receptor levels and proliferation in the V-SVZ. Our data revealed that interactions between RAS receptors play a major role in the regulation of V-SVZ neurogenesis, particularly in proliferation, generation of neuroblasts, and migration to the olfactory bulb, both in young and aged brains, and suggest potential beneficial effects of RAS modulators on neurogenesis.

## 1. Introduction

New neurons destined to integrate into olfactory bulb (OB) circuits are continuously generated from neural stem cells (NSCs) in the ventricular–subventricular zone (V-SVZ) of the lateral ventricles in the adult rodent brain. These self-renewing NSCs exhibit features that are typical of mature astroglial or fetal radial glial cells, such as expression of glial fibrillary acidic protein (GFAP), and produce transit-amplifying neural progenitor cells (NPCs). These NPCs lack self-renewal and divide a few times before they commit to differentiate into doublecortin (DCX)-positive migrating neuroblasts, which migrate anteriorly and terminally differentiate in the OB [1,2]. Neurogenesis is considered a complex multistage process involving proliferation, cell differentiation, and survival/integration into circuitry, finely regulated by a complex interplay between intrinsic and extrinsic factors. Neurogenesis may be broadly divided into two phases: an early phase that includes proliferation, fate commitment, and migration, and a late phase that involves synaptic development and survival of newly born neurons [3]. Additionally, neurogenesis is affected by different conditions such as stress, exercise, brain injury, or aging [4,5]. In fact, aging is associated with structural changes in the V-SVZ and a significant decline in neurogenesis [6,7]. However, the regulatory network underlying neurogenesis and the possible factors linked to its impairment with age remain poorly understood [6,8,9]. Because endogenous NSCs appear to harness the potential to repair lesioned circuits, the identification of signals and mechanisms that could stimulate NSC activity in the elderly is relevant.

The renin–angiotensin system (RAS) has been classically considered a circulating humoral system with fundamental actions on blood pressure regulation and sodium and water homeostasis. The enzyme renin hydrolyses its substrate, angiotensinogen (AGT), to form the decapeptide angiotensin I (Ang I), which is biologically inactive until it is converted to angiotensin II (Ang II), the principal RAS effector, by angiotensin converting enzyme (ACE). Ang II acts through activation of two major receptors, the Ang II type-1 (AT1) and type-2 (AT2) receptors, which usually induce opposite effects [10]. In addition to the “classical” endocrine RAS, local paracrine RAS have been identified in different tissues including the brain, where all the components of a classic SRA are detected and play important functional effects [11]. AT1 receptors activate nicotinamide adenine dinucleotide phosphate (NADPH) oxidase (NOX) and reactive oxygen species (ROS) production in different tissues including the brain [12,13,14], resulting in a pro-oxidative and pro-inflammatory environment. AT2 receptors counteract the deleterious effects of AT1 receptors (i.e., anti-oxidative, anti-inflammatory) through several mechanisms, including downregulation of AT1 expression [15,16]. More recently, new RAS components have been discovered, such as the anti-oxidative, anti-inflammatory Mas receptor (MasR), which adds another level of complexity to the system [17,18].

Expression of AT1 is similar before and after birth, or is even higher in the adult brain. Early descriptions have indicated AT2 receptors are very abundant in fetal tissues in agreement with actions in processes of cell proliferation, maturation, apoptosis, and regeneration [19,20]. Although it was classically considered that the expression of AT2 receptors decreased after birth [21,22], recent methodologies for their detection suggest relevant actions in the adult brain [23,24]. However, the potential role of RAS in adult neurogenesis, its aging-related alterations, and its possible role in regulation of neurogenesis in different neurological diseases has not been thoroughly investigated [25,26,27,28,29]. In the study reported here, we investigated the role of major RAS receptors on neurogenesis, particularly in proliferation, generation of neuroblasts, and migration to the OB, in the V-SVZ of both young and aged rodents.

## 2. Materials and Methods

### 2.1. Animals and In Vivo Treatments

Young (3–4 months old) and aged (20–22 months old) male Sprague-Dawley rats and young (3–4 months old) and old (20 months old) male C57Bl/6J mice (wild type, WT) were used in the present study. Mice carrying genetic deletions of the Agtr1 (AT1-KO) [30] gene were obtained from The Jackson Laboratory (Bar Harbor, ME, USA) and Agt2 mutant mice (AT2-KO) [31] were generously donated by Dr. Daniel Henrion (University of Angers, Angers, France). All experiments were carried out in accordance with the Spanish (RD53/2013) and European Communities Council Directive (2010/63/EU and 86/609/CEE) and were approved by the corresponding committees at the University of Santiago de Compostela and Xunta de Galicia (15005/15/002) and the University of Valencia. Rats and mice were randomly distributed in several experimental groups for the treatments. Some were treated for 15 days with the AT1 receptor antagonist candesartan cilexetil (1 mg/kg/day; AstraZeneca, Madrid, Spain) administered orally in a boluses mixed with “Nocilla” hazelnut-cream spread from Idilia Foods (Valencia, Spain) [32]. Control animals received the similar boluses without the drug. Mice treated with the AT2 receptor agonist C21 (Vicore Pharma, Gothenburg, Sweden) were intraperitoneally (i.p.) injected for 15 days at a dose of 0.3 mg/kg/day of the agonist according to previous studies [33,34,35,36] or an equal volume of vehicle (0.9% saline). At the last day of treatment with the drugs, rats or mice were injected i.p. with four pulses of bromodeoxyuridine (BrdU; 50 mg/kg; Sigma-Aldrich, Merck, Madrid, Spain), one every two hours, and killed 24 h after the last injection. To label BrdU-label retaining cells (BrdU-LRC), mice treated with C21 or vehicle for 15 days were injected repeatedly with seen injections of BrdU, one every 2 h, and allowed to survive for a month [37].

### 2.2. Immunohistochemistry

Animals were deeply anesthetized and perfused transcardially with cold 4% paraformaldehyde in 0.1 M phosphate buffer, pH 7.4. Brains were dissected out and either cryoprotected and cut with a sliding microtome or sectioned with a vibrotome into 40 μm coronal sections. Series of free-floating sections were labelled using colorimetric single immunostaining or double immunofluorescence. For BrdU detection, sections were first denatured by incubation with 2N HCl for 15–30 min at 37 °C and washed with 0.1 M sodium borate buffer (pH 8.9). Sections were preincubated with a blocking solution containing 10% normal serum in potassium phosphate-buffered saline (KPBS) with 1% bovine serum albumin (BSA; Sigma-Aldrich, San Luis, MO, USA) and 0.3% Triton X-100 (Sigma-Aldrich) for 1 h at room temperature (RT). The sections were then incubated overnight at 4 °C with the corresponding primary antibodies: rabbit polyclonal antibody against AT1 receptor (1:100; sc-31181; Santa Cruz Biotechnology, Heidelberg, Germany), goat polyclonal AT2 receptor antibody (1:100; sc-9040; Santa Cruz Biotechnology), mouse monoclonal antibody (1:500; B2531; Sigma-Aldrich), or rat monoclonal antibody (1:800; ab6326; Abcam, Cambridge, UK) to BrdU; goat polyclonal antibody to DCX (1:1,000; sc-8066; Santa Cruz Biotechnology); or mouse monoclonal antibody to GFAP (1:500; MAB360; Millipore, Merck Chemicals, Darmstadt, Germany). The specificity of the AT1 and AT2 antibodies has been established in previous studies [38,39,40] and was also confirmed in our laboratory [23,41]. For immunoperoxidase detection, sections were washed and incubated for 1 h at RT with the corresponding biotinylated secondary antibody diluted 1:500. Sections were then incubated with avidin-biotin-peroxidase complex (ABC complex; 1:150; Thermo Fisher Scientific, Waltham, MA, USA). Finally, the labeling was visualized using 3,3′diaminobenzidine (DAB; Sigma-Aldrich). For immunofluorescent detection, sections were incubated with the corresponding Alexa Fluor-488, Fluor-647, or Fluor-568 secondary antibodies (Molecular Probes, Thermo Fisher Scientific, Waltham, MA, USA) for 2 h at RT. Finally, tissue sections were incubated for 30 min at RT with the DNA-binding dye Hoechst-33342 (3 × 10^−5^ M in KPBS) or 4′,6-diamidine-2′-phenylindole dihydrochloride (DAPI; 1 µg/mL; Sigma-Aldrich), mounted on gelatin-coated slides and coverslipped with Immu-Mount (Thermo Fisher Scientific). Tissue sections were visualized with a Leica AOBS-SP5X (Leica Biosystems, Wetzlar, Germany) or an Olympus FV-10i (Olympus, Barcelona, Spain) confocal laser-scanning microscope.

### 2.3. Cell Counting

Quantification of BrdU-immunoreactive (-ir) or DCX-ir cells was carried out in series of five sections to cover the rat V-SVZ (between Bregma coordinates 1.70 and 0.20 mm), or in series of three sections to cover the mouse V-SVZ (between Bregma coordinates 1.54 and 0.14 mm) [42]. Sampling was carried out with the computer assisted stereological toolbox (CASTGrid system; Computer Assisted Stereological Toolbox; CAST version 2.1.5.9; Olympus, Ballerup, Denmark). The V-SVZ was delineated with a 4× magnification objective. A counting frame of 450 μm^2^ was used for cell sampling, and cell profiles were observed and counted with a 100× oil objective (NA 1.4). The total numbers of BrdU-positive or DCX-positive cells were calculated according to the optical fractionator formula [43]. The V-SVZ volume was estimated according to Cavalieri’s method [44].

### 2.4. Flow Cytometry

After dissection, both V-SVZs from each mouse were minced and enzymatically digested using the Neural Tissue Dissociation kit (T) (#130-093-231; Miltenyi, Bergisch Gladbach, Germany) following the instructions of the manufacturer in a gentleMACS Octo Dissociator with heaters (Miltenyi). Following addition of a trypsin inhibitor (T6522; Sigma-Aldrich) at 100 μg/mL in 3 mL of washing medium (0.6% glucose, 0.1% NaHCO_3_, 5 mM HEPES, 2 mM l-glutamine, 0.4% BSA, 1X antibiotic/antimicotic in DMEM/F-12) and mechanical dissociation by pipetting up and down 20 times through a plastic Pasteur pipette, cell suspension was filtered (40 μm nylon filter), pelleted (300g, 10 min), and incubated with the Dead Cell Removal kit (#130-090-101; Miltenyi) following the instructions of the manufacturer. The eluted living fraction was pelleted (300g, 10 min) and incubated in 100 μL of blocking buffer (HBSS without calcium and magnesium, 10 mM HEPES, 2 mM EDTA, 0.1% glucose, 0.5% BSA) containing the specific primary antibodies for 30 min at 4 °C: CD45-BUV395 (1:200; BD 565967; Becton Dickinson, Madrid, Spain), O4-Biotin (1:30; #130-095-895; Miltenyi), CD31-BUV395 (1:100; BD 740239; Becton Dickinson), Ter119-BUV395 (1:200; BD 563827; Becton Dickinson), Streptavidin-Alexa350 (1:200; s11249; Molecular Probes, Thermo Fisher Scientific), EGF-Alexa488 (1:300; E13345; Molecular Probes, Thermo Fisher Scientific), CD24-PerCP-Cy5.5 (1:300; BD 562360; Becton Dickinson), GLAST-PE (1:20; Miltenyi), and CD9-Vio770 (1:20, #130-102-384; Miltenyi). After washing with 1 mL of blocking buffer (300g, 10 min at 4 °C), labelled samples were analyzed using an LSR-Fortessa cytometer (Becton Dickinson) with 350, 488, 561, and 640 nm lasers. Single cells were gated initially by size (FSC) and complexity (SSC) and then by SSC-A and SSC-H discordance. Prior to NSC and NPC gatings, dead cells and some non-relevant cells were excluded by DAPI (0.1 μg/mL, added just before the analysis; D9542; Sigma-Aldrich), and staining of non-neurogenic cells using well-established markers (CD45, CD31, Ter119, O4) [45,46].

### 2.5. Laser Capture Microdissection (LCM)

Sections containing the mouse or rat V-SVZ were stained with a shortened protocol for neutral red (1% aqueous solution; 2 min at 4 °C). LCM was performed using a PALM MicroBeam system (Zeiss, Oberkochen, Germany) controlled by PALM Robo software [47,48]. V-SVZ cells (or striatal cells, used as a control) were visualized under bright-field microscopy at 40× magnification. Cell pools were selected and then cut and catapulted by laser pulses into an adhesive microtube cap (Zeiss). Cell pools were immediately lysed in RTL lysis buffer (Qiagen, Hilden, Germany) containing β-mercaptoethanol for 30 min at RT and stored at −80 °C.

### 2.6. Neurosphere Cultures

NSCs were isolated from the V-SVZ of the lateral ventricle of adult young (3–4 months old), aged (20 months old) male mice, or young AT1-KO and AT2-KO mice as previously described [49,50]. Primary neurospheres were counted after 7 days in vitro. For passages, neurospheres were mechanically dissociated to a single-cell suspension and reseeded in growth media supplemented with the specific treatments: Ang II (100 nM; Sigma-Aldrich); ZD7155 or candesartan (AT1 receptor blockers; 1 μM; Tocris, Bristol, United Kingdom); PD123319 (AT2 receptor blocker; 1 μM; Sigma-Aldrich); CGP42112A (Sigma-Aldrich) or C21 (AT2 receptor agonists; 1 μM); Ang1-7 (1 μM; Sigma-Aldrich); and A779 (MasR blocker; 1 μM; Bachem, Bubendorf, Switzerland). The doses were selected according to suggestions from previous studies [33,35,51,52,53,54,55]. The diameters of primary and secondary neurospheres were measured after 7 or 5 days, respectively.

### 2.7. RNA Extraction and RT-PCR

In microcaptured mouse and rat cells, total ribonucleic acid (RNA) extraction was performed using the RNeasy Micro kit (Qiagen), and in neurosphere cells was performed using TRIzol (Invitrogen, Thermo Fisher Scientific) according to the manufacturer’s protocol. RNA was solubilized in 14 µl of RNAse-free water and transcribed into complementary DNA (cDNA) using the Moloney murine leukemia virus (M-MLV; 200U; Invitrogen, Thermo Fisher Scientific) reverse transcriptase (RT). For polymerase chain reaction (PCR), a reaction mix containing the BioTaq DNA polymerase (Bioline, London, United Kingdom), NH_4_ reaction buffer, MgCl_2_, dNTPs (Invitrogen, Thermo Fisher Scientific), and the specific primer pairs (Sigma-Aldrich; see Table 1) were used. Primers were designed for each gene using NCBI Primer-BLAST. PCR was performed using a MultiGene Thermal Cycler (Labnet, Madrid, Spain). Finally, the PCR products were separated using agarose gel electrophoresis and were visualized with a UV detection system (Chemidoc XRS System, BioRad, Hercules, CA, USA). The mRNA expression of AT1 and AT2 receptors was determined by real-time quantitative RT-PCR as described in previous studies [56]. Briefly, PCR was performed using a real-time QuantStudio3 platform (Applied Biosystems, Foster City, CA, USA), the EvaGreen qPCR MasterMix (Applied Biological Materials Inc., Vancouver, Canada), and the primer sequences indicated in Table 1. The data were evaluated by the delta Ct method (2^- ΔΔCt^), where Ct is the cycle threshold. Expression of each gene was obtained as relative to the housekeeping (β-actin) transcripts, and the relative amount of mRNA was presented in the form of fold change over control.

### 2.8. Combined HPLC and Western Blot (WB)

AGT was purified and detected in neurosphere growth culture media as previously described [57] with slight modifications. Culture media (1 mL) were collected, heated at 37 °C, and injected (20 µL/injection) into a Shimadzu HPLC system (Shimadzu, Kyoto, Japan). AGT was separated on a reverse phase column (Perkin Elmer Aquapore ODS 300, Perkin Elmer, Madrid, Spain) at 37 °C and a flow rate of 1.5 mL/min by using a two-step separation method consisting in an initial isocratic elution (5% of acetonitrile with 0.1% phosphoric acid for 5min) and then a linear gradient from 5% to 66.5% of acetonitrile with 0.1% phosphoric acid (30 min). The AGT fraction was visualized at 240 nm with a UV-VIS detector (Shimadzu SPD-20 AV, Shimadzu), collected in a fraction collector (Shimadzu FRC-1′A, Shimadzu), and dried in a vacuum concentrator (Savant ISS110, Thermo Fisher Scientific). The pellet was resuspended in RIPA buffer containing a protease inhibitor cocktail (Sigma-Aldrich) and the presence of AGT in the fraction was confirmed by WB analysis. Briefly, proteins were separated using 5%–10% Bis-Tris polyacrylamide gels and transferred to nitrocellulose membranes. The membranes were incubated overnight with a goat primary antibody against AGT (1:200; sc-7419; Santa Cruz Biotechnology) and then for 1 h with the HRP-conjugated secondary antibody donkey anti-goat IgG (1:2500; sc-2020; Santa Cruz Biotechnology). Immunoreactivity was detected with a Lumminata Crescendo HRP Chemiluminescent Kit (Millipore) and visualized with a chemiluminescence detection system (ChemiDoc XRS System, BioRad, Hercules, CA, USA).

AT1 and AT2 protein levels were detected in mouse V-SVZ and striatum (i.e., positive control) by WB analysis (see above) using the corresponding primary antibodies from Santa Cruz Laboratories (1:200): goat polyclonal anti-AT1 (sc-31181) and rabbit polyclonal anti-AT2 (sc-9040), previously validated [41]. GAPDH (rabbit polyclonal antibody; 1:50000; G9545; Sigma-Aldrich) was used as a loading control.

### 2.9. Statistical Analysis

Two-group comparisons were carried out by using Student’s *t*-test. Multiple comparisons were analyzed by one-way analysis of variance (ANOVA) followed by Bonferroni post hoc test. The normality of populations and homogeneity of variances were tested before each analysis of variance. All culture data were obtained from at least three separated experiments and normalized to the values of the control group of the same batch (100%). The results are presented as means ± standard error of the means (SEM). Differences were considered statistically significant at *p* < 0.05. All statistical analyses were performed with SigmaPlot 11.0 (Systat Software Inc., San Jose, CA, USA).

## 3. Results

### 3.1. AT2 Receptors Mediated Promoting Effects of Ang II on Neurosphere Formation

**Figure 1:**
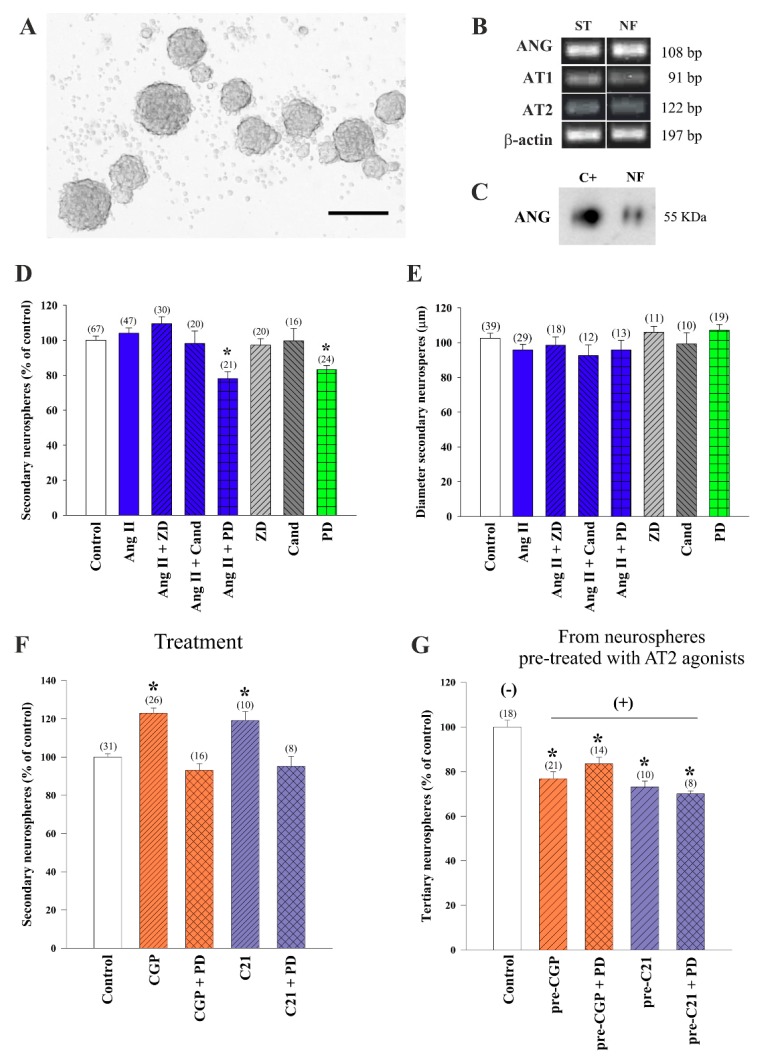
Generation of neurospheres from the ventricular-subventricular zone (V-SVZ) of young mice. (**A**) Photomicrographs showing floating neurospheres obtained from wild-type mice. (**B**) Representative bands for angiotensinogen (ANG), Ang II type-1 (AT1) and type-2 (AT2) receptors, and β-actin obtained by RT-PCR in neurospheres (NF). Homogenates of striatum (ST) were used as a positive control. (**C**) ANG was detected in neurosphere culture medium by HPLC and visualized by western blot. ANG (250 μg/mL) was used as a positive control (C+). Bar graphs showing the number (**D**) and diameter (**E**) of neurospheres after treatment with angiotensin II (Ang II), AT1 receptor antagonist (ZD7155 or candesartan), and AT2 receptor antagonist (PD123319). Histograms showing the number of neurospheres after treatment with AT2 receptor agonists (CGP42112A or C21) and the AT2 antagonist PD123319 ((**F**); treatment) or in cultures derived from neurospheres pre-treated with AT2 agonists and reseeded in the absence of any treatment ((**G**); neurospheres derived from cultures pre-treated with AT2 agonists; pre- = previously treated with). All culture data were obtained from at least three separated experiments. Data are means ± standard error of the mean (SEM). * *p* < 0.05 relative to control (untreated) group (one-way ANOVA and Bonferroni post hoc test.). SEM = standard error of the mean. ANOVA = analysis of variance. bp = base pairs. Scale bar: 150 μm.

As a first approach to the analysis of local RAS in the V-SVZ murine niche, we isolated subventricular cells from young adult mice and plated them as single cells in serum-free growth medium containing mitogens EGF and FGF-2 to produce neurospheres, floating clonal aggregates that can be expanded through subculture (Figure 1A). Neurospheres can be formed by NSCs and NPCs and constitute an ideal system to evaluate the action of signaling pathways in proliferation and self-renewal [49]. The endogenous expression of AGT, AT1, and AT2 receptors by neurosphere cells was confirmed in cell lysates by PCR (Figure 1B). Moreover, the presence of the angiotensinogen (ANG) protein in the medium conditioned by the cells during 5 days was demonstrated by combining HPLC-based extraction and WB [57] (Figure 1C). Because the results indicated the possibility of RAS actions in neurospheres, we next seeded single cells dissociated from neurospheres in medium with and without 100 nM Ang II combined with concurrent administration of AT1 and AT2 receptor antagonists, 1 µM. In particular, we used peptide antagonist ZD7155 (ZD from now on) and non-peptide antagonist candesartan to block AT1 receptors and peptide PD123319 (PD from now on) to block AT2 receptors. AT1 receptor inhibition did not produce any change in the numbers of neurospheres, but we found reduced numbers of neurospheres in the cultures treated with the AT2 receptor inhibitor, independently of the exogenous addition of Ang II, indicating that the AT2 receptor mediated promoting effects of endogenous and exogenously added Ang II in neurosphere formation (Figure 1D). We did not find, however, any significant differences in sphere diameters among treatments, suggesting that AT2 plays a role in neurosphere initiation but not growth (Figure 1E).

To directly test that AT2 receptors indeed promote neurosphere formation, we plated individual neurosphere cells in the presence of two different specific agonists. Treatment with agonist CGP42112A (CGP from now on) induced an increase in the number of spheres generated, which was blocked by the AT2 receptor antagonist PD, and similar results were obtained with the non-peptide AT2 receptor agonist C21 (Figure 1F). To analyze whether the AT2 receptor-mediated positive effects on neurosphere numbers included effects on self-renewal, we re-plated cells obtained from neurospheres that had been grown in the presence of the AT2 receptor agonists and antagonists in growth medium without any specific treatment and evaluated neurosphere formation. In the cultures in which we had withdrawn the AT2 receptor modulators (i.e., derived from neurospheres pre-treated with AT2 agonist; ‘pre-treated’ condition), we could obtain information about the type of cell division that was promoted by the drugs when they were present (‘treated’ condition; Figure 1F) [49]. We observed reduced proportions of new neurospheres formed by cells that had been expanded in their previous passage in the presence of AT2 receptor agonists (Figure 1G). These data suggested that AT2 receptor activation induces asymmetrical division of neurosphere-forming cells, an effect that results in increased numbers of neurospheres that are, however, less clonogenic (i.e., asymmetrical divisions lead to a loss of stemness capacity and reduced ability to generate new neurospheres, i.e., tertiary neurospheres).

### 3.2. AT2 Receptor Agonist C21 Stimulated Activity in the V-SVZ Niche

**Figure 2:**
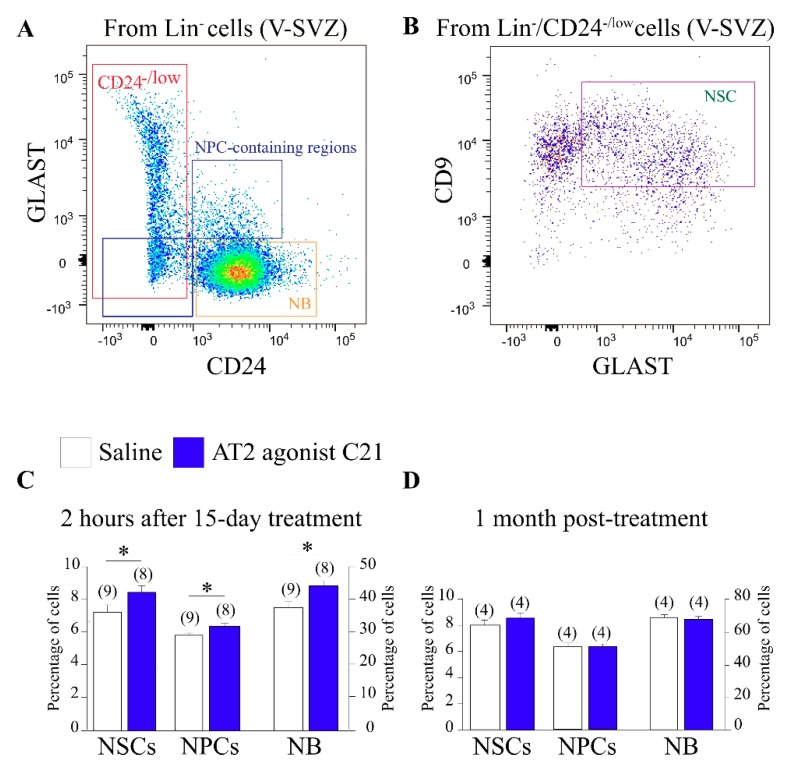
(**A**) FACS analysis of neural stem cell (NSC), neural progenitor (NP), and neuroblast (NB) populations in the ventricular–subventricular zone (V-SVZ) within the Lin- population discriminated by levels of CD24 and GLAST. (**B**) FACS analysis of NSCs within the Lin-/CD24-/low cells discriminated by levels of GLAST and CD9. (**C**) Histogram showing the quantification of NSCs, NP, and NB FACS analyses 2 h after the last dose of 15 days C21 treatment; the levels of these three populations of cells raised up after 15 days of treatment. (**D**) Histogram showing FACS analyses quantification of NSC, NP, and NB populations 1 month after C21 treatment showing no differences between experimental groups. Data are means ± standard error of the mean (SEM). * *p* < 0.05 (Student’s *t*-test).

Our data indicated that AT2 receptor activation induces proliferation of neurosphere-forming cells in the V-SVZ. We, therefore, decided to analyze the in vivo effects of the pharmacological activation of the AT2 receptor using its non-peptide agonist C21. We analyzed the cell composition of V-SVZs dissected from WT mice treated with C21 using combinations of multiple markers and FACS technology [46,58,59,60]. After discarding non-neurogenic cells, the remaining cell pool was sub-divided using a panel of markers that included antibodies to GLAST (astroglial cells), CD24 (neuroblasts), and CD9 (higher in NSCs than in astrocytes), as well as fluorescently-labeled EGF, which labels activated cells within the lineage, as previously described [45]. We classified the cells into EGFR^+^ proliferating and EGFR— postmitotic GLAST—CD24^high^ neuroblasts, EGFR^+^ proliferating NPCs (GLAST—CD24^low^ and GLAST^+^CD24^high^ cells), and CD9^high^GLAST^+^CD24^low/neg^ NSCs [45] (Figure 2A,B). After a 15 day treatment with C21, we found a significant increase in the total population of NSCs and of NPCs (Figure 2C). In line with these data, we observed higher proportions of neuroblasts arriving at the OB (Figure 2C). The C21 effect was transient, as we could not observe any signs of increased activation one month after the treatment (Figure 2D). The data together further indicated that pharmacological activation of the AT2 receptor increased V-SVZ activity, and that the effect was present during a chronic treatment (i.e., 15 days) but did not continue for a long period if the treatment was stopped.

### 3.3. AT1 Receptor-Dependent Restraining Action on Adult Neurogenesis Was Counteracted by Stimulatory AT2 Receptor Activity

**Figure 3:**
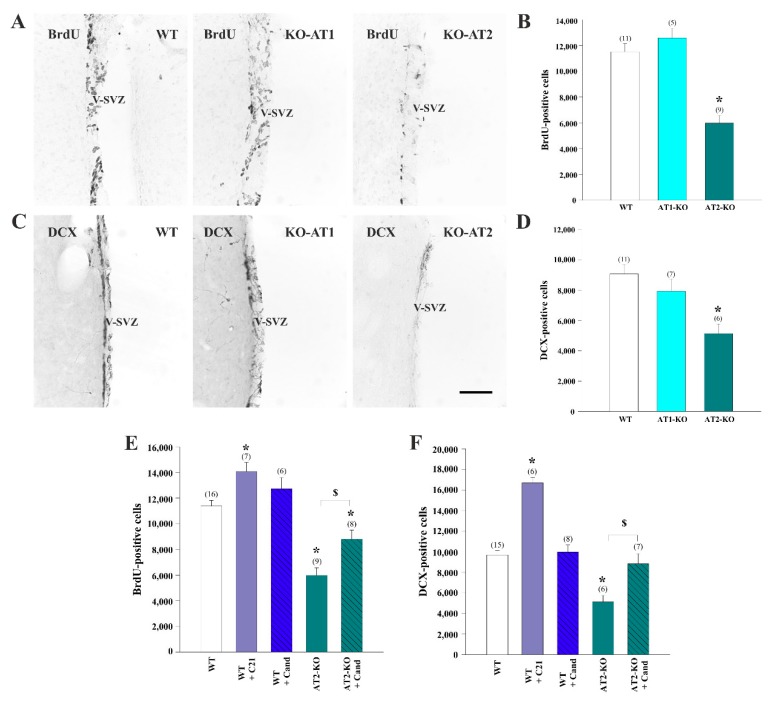
Bromodeoxyuridine (BrdU (**A**,**B**) or doublecortin (DCX)-immunoreactive (-ir) (**C**,**D**) cells in the ventricular–subventricular zone (V-SVZ) of wild-type (WT) mice, AT1 receptor-deficient mice (AT1-KO), and AT2-deficient mice (AT2-KO). Number of BrdU (**E**) and DCX-ir (**F**) cells in the V-SVZ of WT mice, WT mice treated with the AT1 antagonist candesartan (Cand), WT mice treated with the AT2 agonist C21, and AT2-KO mice untreated or treated with candesartan for 15 days. Data are means ± SEM. * *p* < 0.05 relative to WT controls (one-way ANOVA and Bonferroni post hoc test); in figures (**E**,**F**), ^$^ *p* < 0.05 relative to AT2-KO group (Student’s *t*-test). ANOVA = analysis of variance. Scale bar: 200 μm.

Although AT2 receptors appeared to play a significant role in the activation of neurosphere-initiating cells by endogenous Ang II, complex interactions between AT1 and AT2 receptors were observed in different tissues and, therefore, we turned to mice that carry specific deletions in the genes coding for AT1 and AT2 receptors to dissect individual and combined functions in vivo. AT1-KO and AT2-KO homozygous mutant mice and WT littermates were injected i.p. with BrdU (four pulses at 50 mg/kg, every 2 h) and sacrificed 24 h after the last injection to detect proliferating cells. Consistent with our in vitro data, we did not observe any significant change in the number of BrdU-ir and DCX-ir cells in AT1-KO mice, whereas both parameters were significantly reduced in AT2-KO mice relative to control WT mice (Figure 3A–D). These data further indicated that the AT2 receptor is a NSC positive regulator and demonstrated its essential role in vivo.

We next employed the brain-permeable AT1 receptor antagonist candesartan and the AT2 receptor agonist C21. First, we treated young WT mice with the AT2 agonist C21 or vehicle for 15 days, and on the last day of treatment the animals received BrdU as described above. Specific activation of the AT2 receptor with the C21 agonist increased the number of BrdU-ir and DCX-ir cells in the V-SVZ relative to vehicle-treated WT mice, as expected (Figure 3E,F). In line with our previous results, no significant changes were observed when candesartan was administered to WT mice, however, interestingly, the reductions observed in the AT2-KO mice were partially restored by the AT1 receptor inhibitor (Figure 3E,F). These data indicated that AT1 receptors inhibited proliferation in the V-SVZ, and that this inhibitory effect was normally blocked by the AT2 receptor activity (i.e., the AT1 inhibitory effect is mostly blocked by AT2 in basal conditions).

### 3.4. Cross-Regulation of AT1 and AT2 Receptors Underlies Ang II Effects on Neurosphere-Forming Cells

**Figure 4:**
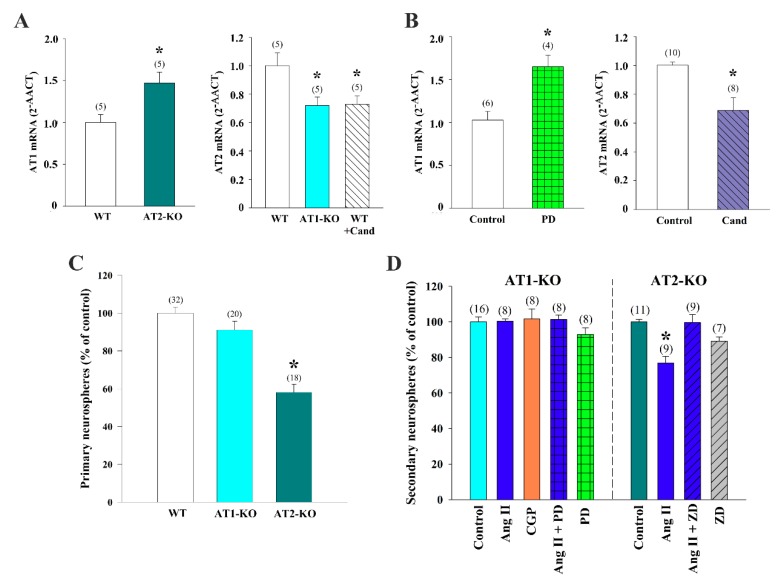
Expression of AT1 and AT2 receptors in the adult ventricular–subventricular zone (V-SVZ) and neurospheres generated from the V-SVZ of mice. (**A**) AT1 receptor expression was higher in the microdissected V-SVZ of AT2 receptor deficient (AT2-KO) mice and AT2 receptor expression was lower in the microdissected V-SVZ of AT1 receptor deficient (AT1-KO) mice and wild-type (WT) mice treated with the AT1 antagonist candesartan. (**B**) AT1 expression was higher in neurospheres treated with the AT2 antagonist PD123319 than in controls, and AT2 expression was lower in neurospheres treated with the AT1 antagonist candesartan than in controls. The number of primary neurospheres obtained from WT, AT1-KO, and AT2-KO mice is shown in (**C**). (**D**) Bar graphs showing the number of neurospheres derived from AT1-KO or AT2-KO mice after the corresponding treatments. All culture data were obtained from at least three separate experiments. Data are means ± SEM. * *p* < 0.05 relative to controls (Student’s *t*-test or one-way ANOVA and Bonferroni post hoc test). Ang II = angiotensin II; CGP42112A = AT2 receptor agonist; ZD7155 = AT1 receptor antagonist.

Because our data suggested complex interactions between AT1 and AT2 receptors in the V-SVZ, and because a number of studies in different cells and tissues have shown reciprocal regulation between AT1 and AT2 receptors [61,62,63], we next set up to evaluate expression levels of these two receptors. We visually selected cells of the V-SVZ in lightly stained brain sections of WT, as well in AT1-KO and AT2-KO mice, which were subsequently isolated by laser-mediated capture. RT-PCR detection of AT1 and AT2 receptors with specific primers (see Table 1) in the cell pools of AT1-KO mice or of WT mice that had been treated with candesartan revealed a significant decrease in *Agtr2* mRNA expression, indicating that AT1 receptor activity upregulates the expression of AT2 receptors in the V-SVZ (Figure 4A). In contrast, cells from AT2-KO mice showed a significant increase in *Agtr1* mRNA expression, indicating that AT2 receptors downregulate expression of AT1 receptors in this niche (Figure 4A). In line with these results, treatment of WT neurosphere cultures with the AT2 antagonist PD increased the levels of *Agtr1* mRNA, whereas the AT1 antagonist candesartan reduced *Agtr2* mRNA levels (Figure 4B).

We then decided to test in vitro the pharmacological conditions used in vivo by using our angiotensin receptor modulators in neurosphere cultures obtained from AT1 and AT2 receptor-deficient mice. We dissected the V-SVZ of WT, AT2-KO, and AT1-KO young mice; obtained homogenates; and seeded equal numbers of isolated cells in growth medium to determine primary neurosphere yield. We found that AT1-KO tissue yielded similar numbers of neurospheres as WT tissue, whereas the samples from AT2-KO produced significantly fewer clones, a further indication of the role of AT2 receptors in mediating the stimulatory effects of Ang II on the V-SVZ (Figure 4C). Despite these changes, no significant differences were detected size of the clones in any of the genotypes (diameter, in μm ± SEM: 106.8 ± 2.8 in WT, 106.0 ± 3.3 in AT1-KO, and 108.8 ± 2.9 in AT2-KO primary spheres). To analyze the effects of Ang II mediated by each receptor, we next plated AT1-KO and AT2-KO cells in regular neurosphere medium with and without 100 nM Ang II and measured the number of clones formed after 5 days. In line with the pharmacological profile shown before, AT1-KO cultures did not respond to exogenously added Ang II; however, inhibition of the AT2 receptor with PD did not result in reduced neurosphere formation as in WT cultures (Figure 4D). Significantly fewer neurospheres were formed in the AT2-KO cultures treated with Ang II, however, interestingly, AT1 receptor antagonist ZD restored neurosphere generation to untreated levels (Figure 4D). These data indicated that AT1 receptor set a basal inhibition of proliferation that was antagonized by the AT2 receptor, likely through its repressive effects on AT1 receptor levels.

The data together suggest that endogenous and exogenously administered Ang II stimulated neurosphere formation through a complex balance of AT1 and AT2 receptor activities. AT2 receptor activation stimulated proliferation, whereas AT1 receptor activation mediated a basal inhibition of Ang II on proliferation that was counteracted by a repressive action of AT2 receptor-dependent signaling on *Agtr1* expression. In turn, AT1 activity stimulated expression of *Agtr2*, generating a negative feedback which set a basal level of Ang II-dependent regulation of neurogenesis.

### 3.5. Stimulatory Effects of AT2 Receptor Activation on Young and Aged NSC Involved MasR

**Figure 5:**
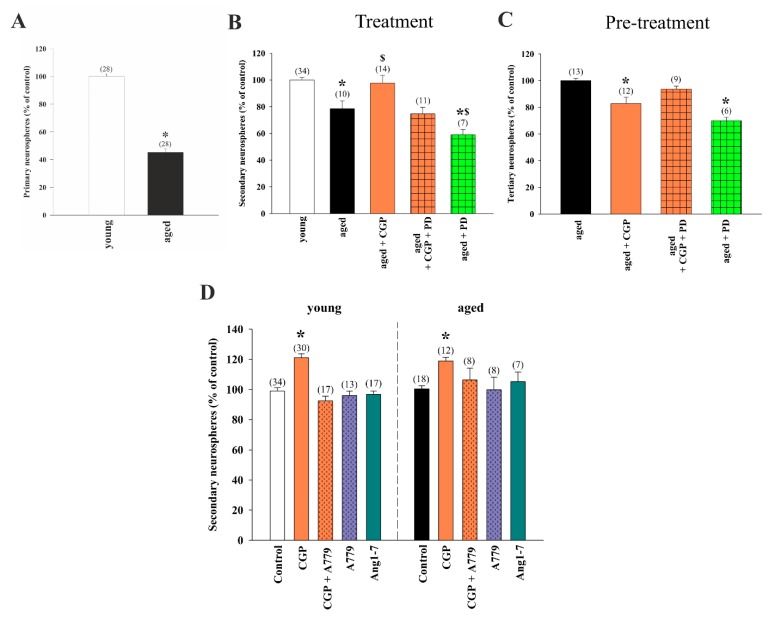
(**A**) Generation of neurospheres from the ventricular–subventricular zone (V-SVZ) of aged mice (20–22 months old) compared to young mice (3–4 months old). Bar graphs showing the number of spheres quantified after treatment with the AT2 receptor agonist CGP42112A and the AT2 receptor antagonist PD123319 ((**B**); treatment), or in cultures derived from neurospheres pre-treated with AT2 agonists and reseeded in the absence of any treatment ((**C**); neurospheres derived from cultures pre-treated with AT2 agonists; pre-: previously treated with). (**D**) Number of neurospheres after treatment with CGP42112A and the Mas receptor antagonist A779 or the Mas receptor agonist angiotensin 1–7 (Ang 1–7). All culture data were obtained from at least three separate experiments. Data are means ± SEM. * *p* < 0.05 relative to control group; in figure (**B**), ^$^ *p* < 0.05 relative to aged group (one-way ANOVA and Bonferroni post hoc test). SEM = standard error of the mean; ANOVA = analysis of variance.

Because activation of the AT2 receptor can stimulate the proliferation of NSCs in young mice, we decided to analyze whether AT2 receptor agonists could also improve the capacity of aged NSCs in generating neurospheres in vitro as a way to evaluate the potential of AT2 activation in elderly mice. In line with previous reports [64], V-SVZs isolated from aged mice yielded reduced numbers of primary neurospheres relative to the young control group (Figure 5A). However, there was no significant difference in neurosphere diameter between groups (in μm ± SEM: 103.4 ± 3.2 in cultures derived from young mice and 100.4 ± 4.3 in cultures derived from aged mice). When primary cultures from elderly mice were subcultured, treatment with the AT2 receptor agonist CGP induced a significant increase in the number of generated spheres, which was similar to that observed in young untreated cultures. This increase was blocked by treatment with the AT2 receptor antagonist PD (Figure 5B). When CGP-treated secondary neurospheres generated from aged mice were reseeded in the absence of any treatment (i.e., neurospheres derived from cultures pre-treated with AT2 agonists; ‘pre-treated’) the number of spheres formed showed a reduction in comparison with control aged group (Figure 5C), indicating that, as in young cultures, the increase in proliferation was accompanied by reduced self-renewal.

Several new components of RAS, such as elements of the Ang 1-7/MasR axis, have recently been discovered [11]. Different data have suggested the cooperative role of MasR with the AT2 receptor in RAS neuroprotective actions [55]. However, the role of Ang 1-7/MasR components in V-SVZ neurogenesis has not been investigated. To address this issue, we treated neurospheres from young and aged mice with Ang 1–7 and with the antagonist of the anti-inflammatory MasR A779. Treatment with Ang 1–7 or with the MasR inhibitor by itself did not significantly affect the number of neurospheres generated in any of the two age conditions. Interestingly, the A779 antagonist prevented the CGP-induced increase in the number of spheres both in young and aged cultures (Figure 5D), suggesting potential interactions between AT2 and MasR receptors.

### 3.6. AT1 Receptor Involvement in the RAS Actions on Adult Neurogenesis in Rat

**Figure 6:**
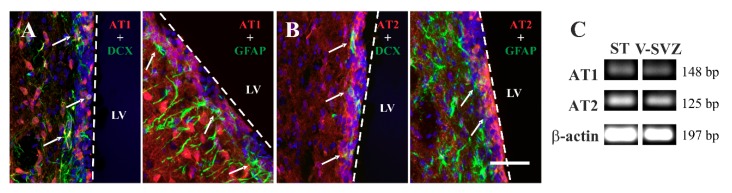
Immunofluorescence labelling for AT1 ((**A**); red) and AT2 ((**B**); red) receptors showing their presence in DCX-positive cells (green) and glial fibrillary acidic protein (GFAP)-positive cells (green) in the rat ventricular–subventricular zone (V-SVZ). Nuclei have been labelled with Hoechst-33342 (blue). (**C**) Representative bands of AT1 and AT2 receptors and β-actin by RT-PCR in rat laser-microdissected V-SVZ. Microdissected cells obtained from the striatum (ST) were used as a positive control. LV = lateral ventricle; DCX = doublecortin. bp = base pairs. Scale bar: 100 μm.

**Figure 7:**
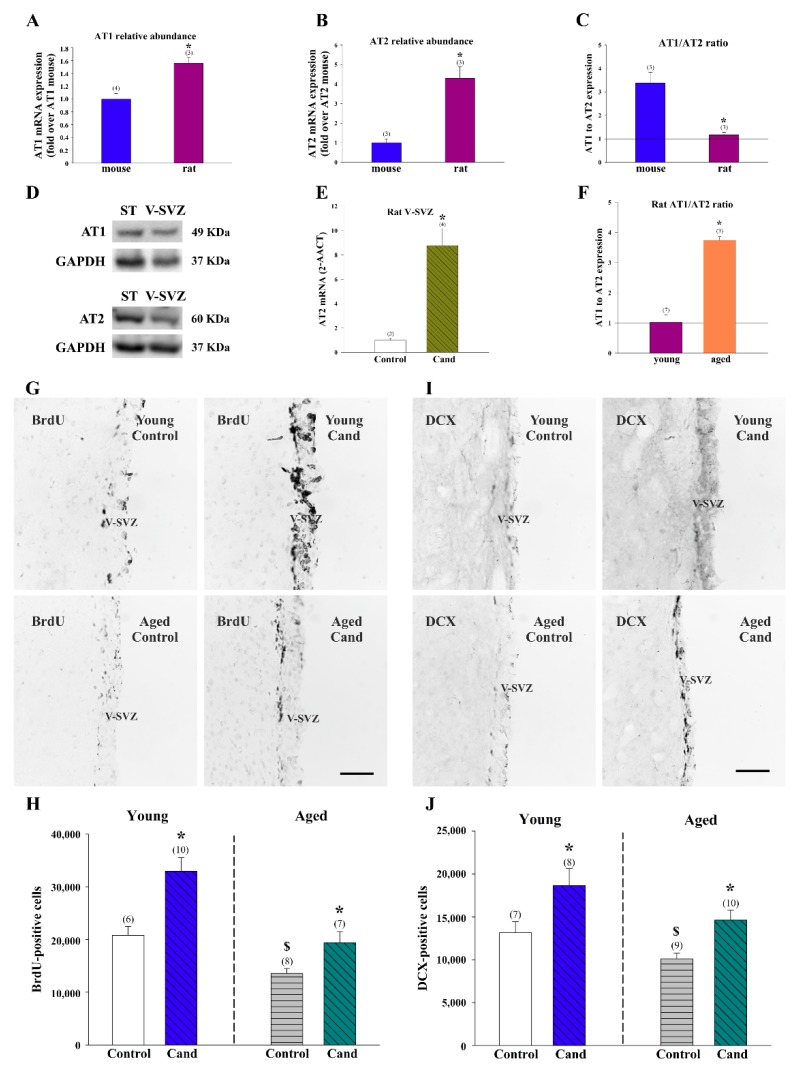
Expression of AT1 and AT2 receptors in the adult ventricular–subventricular (V-SVZ) of rats and mice. The level of mRNA of AT1 (**A**) and AT2 (**B**) was higher in rats than in mice. However, the ratio of AT1/AT2 receptors in the microdissected V-SVZ was higher in mice than in rats (**C**). (**D**) Representative bands of AT1 and AT2 receptors in the mouse V-SVZ detected by western blot. Striatum (ST) was used as a positive control. (**E**) AT2 receptor expression was higher in rats treated with the AT1 receptor antagonist candesartan (Cand) than in control rats. (**F**) The ratio of AT1/AT2 was higher in aged than in young rats. Representative photomicrographs of coronal sections immunostained for BrdU (**G**) and DCX (**I**) of young and aged rat V-SVZ. The estimated number of BrdU and DCX-ir cells in the V-SVZ of the experimental groups is shown in (**H**,**J**). Data are means ± SEM. * *p* < 0.05 relative to the control group of the same age (Student’s *t*-test); in figures (**H**,**J**), ^$^ *p* < 0.05 relative to the control young group (Student’s *t*-test). BrdU = bromodeoxyuridine; DCX = doublecortin; SEM = standard error of the mean. Scale bar: 100 μm.

We next analyzed expression of Ang II receptors in young adult rats. Detection with specific antibodies revealed immunoreactivity for AT1 and AT2 receptors in cells of the adult rat V-SVZ (Figure 6A,B). Double-immunofluorescence detections indicated that most DCX-ir neuroblasts had AT1 and AT2 receptors and that some scattered GFAP-ir cells, likely representing NSCs and/or non-neurogenic astrocytes, were also positive for both receptors (Figure 6A,B). The presence of AT1 and AT2 receptors in the rat V-SVZ was confirmed by RT-PCR (Figure 6C). RT-PCR detection with specific primers in cell pools isolated by LCM from the V-SVZ of young adult rats revealed expression of both receptors at higher levels than in mice (Figure 7A,B). However, the AT1/AT2 ratio was around one in young rats, whereas mouse tissue had three times more AT1 than AT2 receptor mRNA levels (Figure 7C). WB analysis showed the presence of AT1 and AT2 receptor proteins in the mouse V-SVZ (Figure 7D). Furthermore, we observed that rats treated with the AT1 receptor inhibitor candesartan showed a marked increase in *Atgr2* expression in the V-SVZ (Figure 7E), which is consistent with our previous observations in the striatum of candesartan-treated rats [63] but opposite to our observations in the murine V-SVZ (Figure 4A,B). Interestingly, we observed that the V-SVZ of aged animals showed an increase in AT1/AT2 ratio relative to young controls (Figure 7F), which is consistent with our previous observations in the striatum and substantia nigra of aged rats and mice [62,65,66]. The present data indicate the possibility that RAS manipulations (e.g., candesartan) could exert different effects on adult neurogenesis in different species (e.g., rats and mice).

To study the potential role of endogenous Ang II on adult neurogenesis in the rat V-SVZ, we first orally-treated young and aged rats with the AT1 antagonist candesartan or vehicle for 15 days [32]. Due to its non-peptide nature, candesartan efficiently permeates the brain where it can block effects of Ang II at doses that have little effect on blood pressure [67]. At the last day of treatment, rats were injected i.p. with the traceable nucleoside BrdU (four pulses at 50 mg/kg, every 2 h) and sacrificed 24 h after the last injection to detect proliferating cells. We found a significant increase in the number of BrdU-ir cells in the V-SVZ of young animals treated with candesartan relative to vehicle-treated control animals (Figure 7G,H). As previously observed [9], the overall number of BrdU-ir cells was lower in untreated aged animals than in young animals, but they were increased by the treatment with candesartan compared to untreated animals of equivalent age (Figure 7G,H). Similar results were obtained both in young and aged rats when the number of DCX-ir neuroblasts was analyzed (Figure 7I,J). These results indicated that the AT1 receptor restrains proliferation and AT2 receptor expression in the rat V-SVZ. Together, our work demonstrates effects of Ang II on the V-SVZ niche under physiological conditions in both rats and mice, but highlights that the actions of the RAS in adult neurogenesis may be mechanistically different depending on the species under study.

## 4. Discussion

**Figure 8:**
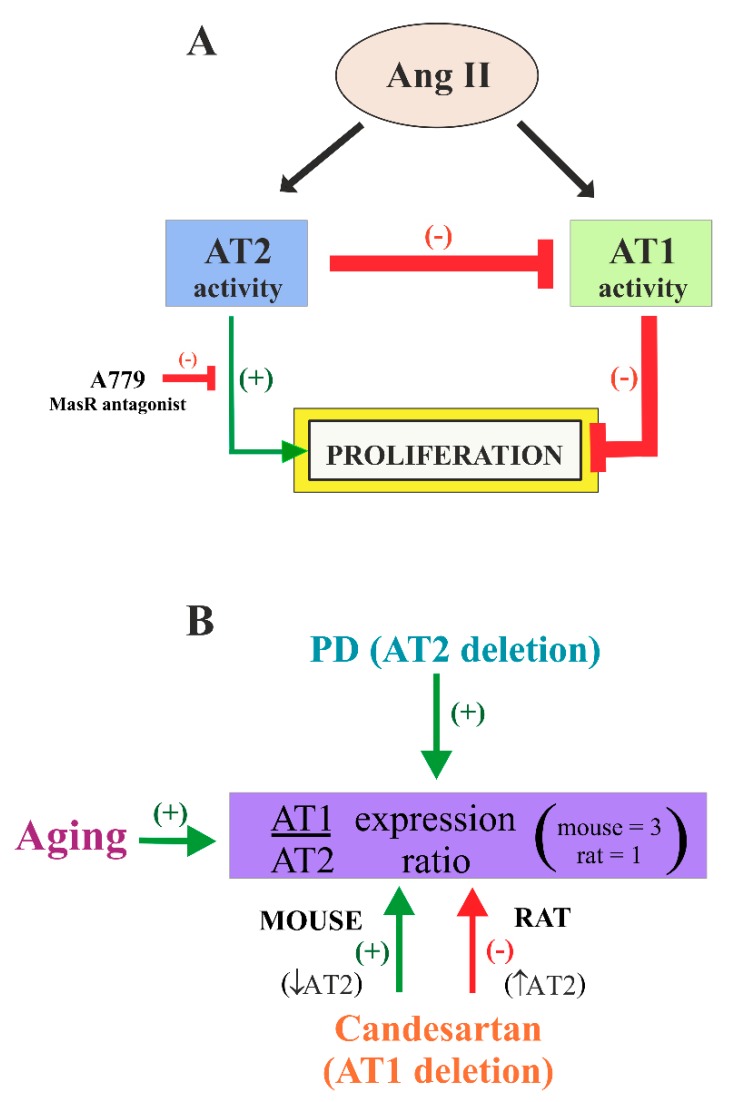
(**A**) Model of the role of angiotensin II (Ang II) AT1 and AT2 receptor activity on the proliferation of V-SVZ cells. Ang II, via AT1 receptors, set a basal inhibition of proliferation that was antagonized by the AT2 receptors, which set a basal level of Ang II-dependent regulation of neurogenesis. In addition, AT2 receptor activity may exert an additional direct stimulatory effect on proliferation, which was modulated by Mas receptors (MasR). (**B**) Aging, candesartan (or AT1 deletion), and PD (or AT2 deletion) regulate AT1/AT2 expression ratio. Aging and PD (or AT2 deletion) increased ratio and candesartan (or AT1 deletion) increased ratio in mice (i.e., decreased AT2 expression) and decreased ratio in rats (i.e., increased AT2 expression). Candesartan = AT1 receptor antagonist; PD123319 = AT2 receptor antagonist.

In the present study, we have shown the presence of AT1 and AT2 receptors in the adult rat and mouse V-SVZ, and in mouse V-SVZ-derived stem/progenitor cells grown as neurospheres. We observed that both AT1 and AT2 receptors were involved in regulation of proliferation of V-SVZ cells (Figure 8A), and that the expression of AT1 and AT2 receptors (i.e., AT1/AT2 ratio) may be modified by aging, drugs such as AT1 receptor antagonists (candesartan), or species differences (Figure 8B). The results are consistent with previous studies showing immunoreactivity for AT1 and AT2 receptors in aggregates of precursor cells obtained from developing brain tissue [19], and with a time-dependent increase in AT1 and AT2 receptor expression in cultured rat NSCs derived from embryonic and adult hippocampus [68,69]. Using confocal microscopy, we observed that AT1 and AT2 receptors were mostly expressed in V-SVZ neuroblasts. Moreover, some GFAP-positive cells, mainly located in the zone lining the striatum, showed co-expression of both receptors. On the basis of this location, these cells may probably correspond to non-neurogenic astrocytes, also called niche astrocytes or type-B2 cells [70]. In addition, the presence of AT1 and AT2 receptors in our cell culture system suggests that both receptors were located on NSCs or type-B1 cells, which were also positive for GFAP, as well as their transit-amplifying progenitors that can form neurospheres [71]. The present results also show that AGT, which is the precursor molecule for Ang II, was released from NSC/precursor cells, as the effects were also observed in the absence of Ang II administration. In the brain, astrocytes are the major source of AGT, and NSC/precursor cells have characteristics of astrocytes [72,73].

The results also show that AT1 and AT2 receptors played a major role in NSC proliferation and/or generation of neuroblasts in the V-SVZ. Most of known actions of Ang II in the adult brain were mediated by AT1 receptors [74]. Local Ang II, via AT1 receptors, has been shown to enhance oxidative stress damage and neuroinflammation contributing to the progression of neurodegeneration [75,76]. However, effects of AT1 on cell proliferation and neurogenesis are controversial. Ang II, through AT1 receptors, is a major activator of the NADPH-oxidase complex, which is a primary source of ROS [77], and enhanced ROS generation plays a recognized role in cell death and pathogenesis. However, previous studies have also shown that ROS derived from NADPH-oxidase activation contribute to the self-renewal [78] and lineage determination in NSCs [79], and that AT1 receptors have an important role in progenitor cell proliferation [80]. Other studies have observed that blockage of AT1 receptors with different antagonists such as candesartan, valsartan, or losartan induced proliferation and neurogenesis in the dentate gyrus [25,26,81], but also that treatment with AT1 antagonists or angiotensin converting enzyme inhibitors diminishes neurogenesis [27,28,82,83,84], or that AT1 blockade does not affect hippocampal neurogenesis [85,86]. There are also controversial results on the effects of AT2 receptors. Activation of AT2 receptors with the selective agonist CGP reportedly induced an increase in BrdU-positive cells in models of traumatic brain [87,88] and proliferation of hippocampal NSCs in vitro [68]. However, antiproliferative effects of AT2 receptors have also been suggested [89,90,91].

Our results suggest that most of the aforementioned controversial results may be explained by the complex interactions between AT1 and AT2 receptors and possible differences in the AT1/AT2 ratio in different regions and species. In AT2-KO mice, which show increased expression of AT1 in the V-SVZ, we observed a marked decrease in proliferation and generation of neuroblasts, which was inhibited by treatment with the AT1 antagonist candesartan, suggesting that a decrease in AT2 activity and an increase in AT1 activity inhibit proliferation and generation of neuroblasts. This was confirmed by treating WT mice with the AT2 agonist C21, which increased proliferation and generation of neuroblasts. Interestingly, AT1-KO mice or WT mice treated with the AT1 blocker candesartan, which also showed downregulation of AT2 receptors, did not show significant changes in proliferation and generation of neuroblasts. A basal inhibition of proliferation by AT1 receptors without any direct effect of AT2 receptors (i.e., beyond inhibition of AT1 activity) should lead to higher levels of proliferation in AT1-KO mice relative to WT mice. These similar levels may be explained by an additional direct positive effect of AT2 receptors on proliferation, which is inhibited by the downregulation of AT2 receptors observed in AT1-KO and candesartan-treated mice or neurosphere cultures. In summary, both downregulation of AT1 and upregulation of AT2 contribute to the increase in V-SVZ proliferation and generation of neuroblasts.

The results obtained in cultures of neurospheres were consistent with that observed in mice. Activation of AT2 receptors led to an increase in the generation of neurospheres. On the contrary, inhibition of AT2 led to a decrease in generation of neurospheres, and no significant changes were observed after blocking AT1 (which also leads to AT2 downregulation in neurospheres) as observed in neurospheres from AT1-KO mice or candesartan or ZD-treated WT mouse neurospheres. We also observed that the generation of neurospheres was reduced after previous stimulation of AT2 receptors, suggesting a possible loss of stemness.

The rat V-SVZ and mouse neurospheres from aged animals showed a significant decrease in proliferation and neurogenesis, which was inhibited by AT2 agonists or AT1 antagonists and exacerbated by AT2 antagonists. These results are consistent with the aforementioned mechanisms, as we observed an increase in AT1/AT2 expression ratio in the V-SVZ of aged animals relative to young controls, as previously observed in different types of cells [92], and particularly in the striatum and substantia nigra [62,65,66]. The present results are consistent with previous studies showing that neurogenesis continues throughout life, although its rate declines with increasing age in rodents and non-human primates [64], and with previous studies showing a progressive reduction in the production of progenitors throughout the mouse adult life and a maintenance of NSC pools [6,7,93]. Interestingly, our results reveal that the reduction in proliferation and generation of neuroblasts and neuropheres observed in the V-SVZ of aged animals may be counteracted by acting on the local RAS, suggesting that old NSCs show proliferation and differentiation capacity after adequate stimulation, which is consistent with other recent observations [94].

In cultures of neurospheres, we also investigated a possible role of Ang1-7/MasR. We administered Ang 1–7 to cultures derived from young and aged mice, and no significant change was detected. However, treatment of cultures with the MasR antagonist A779 blocked the AT2 agonist-induced increase in the number of spheres. This suggests a functional dependence between AT2 and MasR for the AT2-induced generation of neurospheres. Accumulating evidence shows that both receptors are co-localized, form heterodimers, and have different functional interactions in different cells and experimental conditions [27,95,96,97], and recent data from our laboratory (unpublished) showed that young AT2-KO mice show a reduced expression of MasR in the substantia nigra and striatum, which may also contribute to the decrease in proliferation and neurogenesis observed in the V-SVZ of AT2-KO mice. Therefore, although RAS receptors have been associated with different cellular and physiological responses, their role in neurogenesis is complex, as shown by the present results that reveal the presence of AT1, AT2, and MasR interactions possibly both in physiological or pathological situations.

## 5. Conclusions

The present data revealed that the RAS played a major role in the regulation of adult neurogenesis both in young and aged brains. Ang II, via AT1 receptors, set a basal inhibition of V-SVZ proliferation that was antagonized by AT2 receptors. In addition, AT2 receptor activity may exert an additional direct stimulatory effect on proliferation. AT1/AT2 expression and ratio were affected by aging, which resulted in a decrease of neurogenesis. However, AT1 and AT2 receptor expression and neurogenesis may be modulated by therapies with agonists and antagonist of these receptors. Furthermore, RAS may interact with other modulators of neurogenesis. It is well-known that dopamine modulates neurogenesis in the V-SVZ [98], and counterregulatory interactions between Ang II and dopamine receptors have been demonstrated in the striatum [57,66] and other tissues [99,100]. Therefore, it seems plausible that dopamine regulates proliferation and neurogenesis by modulating RAS activity. Our results also suggest the potential beneficial effects of RAS modulators on neurogenesis. However, AT1/AT2 expression, ratio, and responses to drugs may vary depending on the species. Future studies are required to further clarify the effects of manipulation of the brain RAS in humans and pathological conditions associated with neurogenesis impairments, and to provide a solid basis for the use of RAS manipulation in such diseases.

## Figures and Tables

**Table 1:** Primer sequences used for reverse transcription polymerase chain reaction (RT-PCR).

	Primer	Accession Number	Forward Sequence (5′–3′)	Reverse Sequence (3′–5′)
Rat	AT1R	NM_030985.4	GTTAAGGGCCATTTTGTTTTTCTGG	TTGTCTGGATAAATCACACAACCC
	AT2R	NM_012494.3	AACATCTGCTGAAGACCAATAG	AGAAGGTCAGAACATGGAAGG
	β-act	NM_031144.3	TCGTGCGTGACATTAAAGAG	TGCCACAGGATTCCATACC
Mouse	Angiotensinogen (ANG)	NM_007428.4	CTGCTGGCTGAGGACAAG	CGAGGAGGATGCTATTGAGAA
	AT1R	NM_177322.3	CTCTGCTGCTCTCCCGGACTTAA	AGGGCCATTTTGCTTTTCTGGGT
	AT2R	NM_007429.5	CTGGCAAGCATCTTATGTAGTTC	CAAGCATTCACACCTAAGTATTCA
	β-act	NM_007393.5	TCGTGCGTGACATTAAAGAG	TGCCACAGGATTCCATACC
